# Women's Views on Their Diagnosis and Management for Borderline Gestational Diabetes Mellitus

**DOI:** 10.1155/2015/209215

**Published:** 2015-02-16

**Authors:** Shanshan Han, Philippa F. Middleton, Tanya K. Bubner, Caroline A. Crowther

**Affiliations:** ^1^Australian Research Centre for Health of Women and Babies (ARCH), The Robinson Research Institute, Discipline of Obstetrics and Gynaecology, The University of Adelaide, Adelaide, SA 5006, Australia; ^2^Liggins Institute, The University of Auckland, Auckland 1023, New Zealand

## Abstract

*Introduction*. Little is known about women's views relating to a diagnosis of borderline gestational diabetes mellitus (GDM) and the subsequent management. This study aimed to explore women's experiences after being diagnosed with borderline GDM, their attitudes about treatment, and factors important to them for achieving any lifestyle changes. *Methods*. We conducted face-to-face, semistructured interviews with women diagnosed with borderline GDM. *Results*. A total of 22 women were interviewed. After a diagnosis of borderline GDM, 14 (64%) women reported not being concerned or worried. Management of borderline GDM was thought by 21 (95%) women to be very important or important. Eighteen (82%) women planned to improve their diet and/or exercise to manage their borderline GDM. The most frequently mentioned enabler for achieving intended lifestyle change was being more motivated to improve the health of their baby and/or themselves (15 women). The most frequent barrier was tiredness and/or being physically unwell (11 women). *Conclusions*. A diagnosis of borderline GDM caused some concern to one-third of women interviewed. The majority of women believed managing their borderline GDM was important and they planned to improve their lifestyle. Women's own and their babies' future health were powerful motivators for lifestyle change.

## 1. Introduction

Gestational diabetes mellitus (GDM) is defined as glucose intolerance or hyperglycemia with onset or first recognition during pregnancy [1]. GDM is one of the most common complications of pregnancy, with prevalence varying between 1% and 14% around the world [2]. The prevalence of GDM continues to increase in line with the increasing prevalence of maternal obesity and type 2 diabetes mellitus (T2DM) [3–5].

Maternal pregnancy hyperglycemia has a continuous relationship with adverse pregnancy outcomes, including infant being large for gestational age, neonatal hypoglycemia, preterm birth, shoulder dystocia, caesarean section, and preeclampsia [6, 7]. The health risks associated with pregnancy hyperglycemia not meeting GDM diagnostic criteria (borderline GDM) have been found to be similar to those for GDM [8]. Although GDM usually resolves after birth, up to 50% of women with a history of GDM will develop T2DM within 10 years of the index pregnancy [9].

Behavioral management, involving dietary and lifestyle interventions, has been found to be beneficial and is recommended as the primary therapeutic strategy for managing pregnancy hyperglycemia [8, 10]. In-depth understandings of psychosocial factors that determine an individual's behavior are therefore important in the development of tailored lifestyle interventions for women with pregnancy hyperglycemia. This may greatly improve the effectiveness of the care provided [11, 12].

Evidence from previous questionnaire-based studies suggests that women with a positive oral glucose challenge test (OGCT) but a normal oral glucose tolerance test (OGTT) were less likely to perceive their health as “excellent” when compared with women with normal glycemia during pregnancy [13, 14]. However, little is known about their views towards receiving lifestyle management advice or about factors that may influence their ability to make behavioral changes.

This face-to-face, semistructured interview study was nested within the IDEAL randomized controlled trial investigating the effect of dietary and lifestyle advice on women with borderline gestational diabetes [15]. Our study aimed to explore women's experiences after being diagnosed with borderline GDM and their attitudes towards management and to identify factors important to them in achieving any intended lifestyle changes. Borderline GDM was defined as a positive 50 g OGCT (1 hour venous plasma glucose ≥7.8 mmol/L) followed by a normal oral 75 g OGTT (fasting venous plasma glucose <5.5 mmol/L and a 2 hour glucose <7.8 mmol/L) [15].

## 2. Materials and Methods

### 2.1. Participants and Procedure

Women were eligible if they were participants in the IDEAL study [15] and able to communicate in English and within two weeks after trial entry or less than 12 months postpartum. Women who had a history of GDM or developed GDM or were diagnosed with T2DM during the study period were not eligible for this study. Women were recruited either face-to-face or via the telephone using a purposive sampling method at the Women's and Children's Hospital (WCH), Adelaide, Australia, a Level 3 teaching hospital. During the recruitment process, women were made aware that the interview was not for assessment of their knowledge or skill and would not affect their care by attending clinical team. They were advised that information collected during the interview would be kept confidential and anonymous. We aimed to recruit between 16 and 20 women for the interview, to reach data saturation in the thematic analysis when no further new themes or subthemes would be revealed [17].

### 2.2. The Interview

Semistructured, face-to-face interviews, to facilitate a deeper understanding [18], were conducted by a single researcher (SH) with training in interview skills. Interviews were conducted in a quiet office away from the busy hospital clinic area. No explicit time restraints were applied, with each interview typically taking about 25 minutes.

A semistructured question list was prepared and pilot-tested before the interview. The topics were designed to explore the woman's feelings and experiences about a diagnosis of borderline GDM and its subsequent management, as well as factors that might impact on their ability to change behaviors. By the end of each interview, a brief summary of the interview was given to the women by the interviewer to check if anything significant had been missed or if there was any misinterpretation.

#### 2.2.1. Semistructured Question List Used in the Interviews

1. What were your first impressions when you were told that you had borderline GDM?
2. How important do you think it is to provide management for borderline GDM? (Scale: very important, important, not sure, and not very important)
3. Besides the information provided by the IDEAL study, did you seek other information about managing borderline GDM?
4. Since you have been involved in the IDEAL study, have you thought about making some changes to your diet or exercise to improve health?
5. What changes in your diet or exercise did you try and continue with?
6. What helped you achieve the success?
7. What changes did you try but could not continue?
8. What factors made it hard to continue?
9. Is there anything that could help?

### 2.3. Analysis

Each interview was audio-tape-recorded and transcribed verbatim by two people not involved in the study. Field notes and interview summaries were prepared immediately after each interview by the interviewer to help later analysis.

The transcripts of the interviews were analyzed using content analysis methods based on Graneheim and Lundman [19]. To satisfy reliability criteria, the interview transcripts were read and coded by two investigators (SH and TB) independently. Any discrepancies on categorization were solved by discussion and/or consultation with a third independent investigator.

Transcribed data for the different interviews were analyzed thematically by systematic comparisons, derived from grounded theory methods [20], and were organized by themes. Themes were then coded into categories. Data about enablers and barriers for women to achieve their intended lifestyle changes were coded into categories based on the behavioral change wheel framework [21]. Within this framework, the three factors of capability, opportunity, and motivation are considered to be key determinants of an individual's behavior [21]. Capability refers to the individual's psychological and physical capacity to engage in the activity concerned, which includes having the necessary knowledge and skills. Opportunity is defined as all the physical and social factors that make the behavior possible or prompt the adoption of behaviors. Motivation includes all those brain processes that direct behavior, which include habitual processes, emotional responding, and analytical decision-making. Reporting of this study was based on the COREQ (consolidated criteria for reporting qualitative research) guideline [22].

### 2.4. Ethics

This study received approval from the Children, Youth and Women's Health Service (CYWHS) Human Research Ethics Committee (REC 1860/8/12). Written consent from participants was obtained prior to the interviews.

## 3. Results

### 3.1. Participants

**Figure 1:**
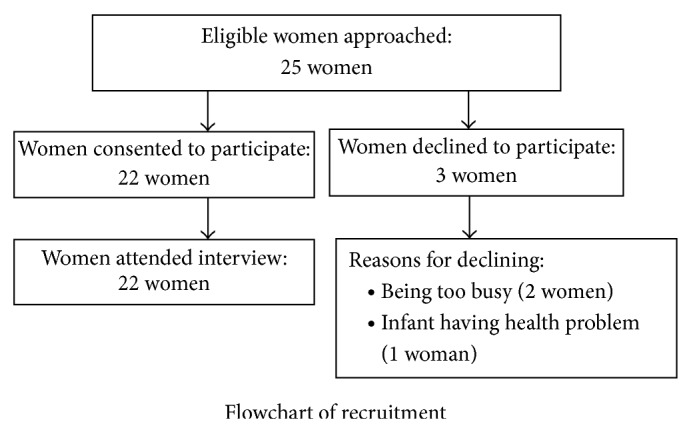


During the study period, 25 eligible women were approached to participate in an interview, of whom 22 provided written informed consent and attended the interview and three women declined to participate. Two women declined because they were too busy and one because of concern about her baby's health (Figure 1).

Of the 22 women who attended interview, 11 (50%) women attended interviews between 31- and 38 weeks gestation and the remaining 11 (50%) women attended interviews between four and seven months postpartum. Data saturation was reached within the sample size of 22 women.

Over two-thirds of interview participants were primiparous; two-fifths of women were overweight or obese in early pregnancy; nearly half the women had a family history of diabetes mellitus, one woman had a medical history of hypertension; and over two-thirds of women had a socioeconomic status ranking of average or advantaged (Table 1). All women who attended an interview reported they felt safe and relaxed during the interview.

### 3.2. Women's Reactions to Being Diagnosed with Borderline GDM

Women stated a variety of reactions after being informed that they had borderline GDM (Table 2). For the 14 women (64%) who reported that they were “not surprised,” were “not worried,” or “felt ok” about the diagnosis, nine (64%) gave a reason for not being worried or surprised and five (36%) did not. Three (14%) women reported they were not surprised as they had experienced the same situation of having a positive screening test for GDM followed by a negative diagnostic test in previous pregnancies.

*“Actually with my first daughter, I had the same problem, and that's you know, why I expected that my sugar level could be high with this one as well. So I wasn't quite surprised.” (Woman 3)*

Two (9%) women reported they were not surprised, as they had not been feeling well during pregnancy, which led them to expect a diagnosis of GDM. One woman was not worried following an explanation about borderline GDM.

Three (14%) women reported they were worried and/or had a feeling of failure after learning they had a positive OGCT; however, after being told their OGTT results, they were relieved or no longer felt worried.

*“Definitely felt surprised and a bit like a failure, that I had done something wrong. But, coming back as borderline gestational diabetes wasn't such an issue as having full-blown diabetes…and I don't worry about it.” (Woman 18)*

Eight women (36%) reported being mildly worried or scared about having borderline GDM. The reasons they gave included being unsure about what caused the condition, about the health risks, and about what was an appropriate diet to help reduce the health risks.

*“When I know I [have] the borderline, actually I am scared. Because I always scared my baby will be too big, very hard to deliver, maybe we need to go to caesarean.” (Woman 22)*

### 3.3. Women's Attitudes towards Managing Their Borderline GDM

Almost all of the women (95%) rated managing their borderline GDM as important or very important whilst one woman (5%) was unsure. The most frequent reason given was that they believed management of borderline GDM could help with reducing their health risks or those of their babies.

### 3.4. Information Seeking and Plans for Diet and Exercise

When asked whether they had sought additional information about managing borderline GDM, 11 (50%) women reported they had, while the remaining 11 (50%) women did not. Sources which they used included the Internet (7 women), family members who had a history of T2DM or GDM (5 women), and health professionals (3 women). Four of these 11 women used more than one source for additional information.

For the 11 (50%) women who did not seek additional information, nine of them gave varied reasons that included already having received additional information via the IDEAL trial (4 women), not being worried about borderline GDM (3 women), and having no time to search for information (1 woman). Three women did not offer any reasons for not accessing information.

Thirteen women (59%) planned to improve both their diet and exercise pattern after learning about their borderline GDM diagnosis. Four women (18%) planned to improve diet only and one woman intended to improve exercise only as she felt her dietary pattern was already appropriate. The remaining four women (18%) did not have any plans for changing their diet or exercise patterns, three of them because they felt these were already healthy.

### 3.5. The Influence of Family History of Diabetes Mellitus on Women Feelings and Experiences

Six of the 10 women who had a family history of diabetes mentioned this during their interview. Four women mentioned their family history of diabetes when asked about their feelings after knowing of their borderline GDM. Of these women, three reported they were mildly concerned and one woman reported she was not surprised. Two additional women mentioned their family history of diabetes when asked about information seeking and their plans for diet and lifestyle changes. The remaining four women did not mention their family history of diabetes mellitus during their interview.

### 3.6. Enablers and Barriers for Women to Achieve Intended Diet and Exercise Changes

Enablers and barriers for women to achieve their intended lifestyle changes were classified under the three categories of “capability,” “opportunity,” and “motivation” [21]. Six themes, including physical capability, psychological capability, physical opportunity, social opportunity, automatic motivation, and reflective motivation, were used in our study (Table 3).

#### 3.6.1. Enablers Identified by Women

*Capability.* With physical capability, improved physical health over time was raised as an enabler for both diet and exercise by three women without prompting.

*“…Because I felt better. I had a headache every single day for about a month, and as soon as I cut out a lot of the simple sugars the headaches went away and that was enough incentive to not ever, just not have any more.” (Woman 1) *

*“I've hired a cross-trainer; I just was waiting until I was all good down my caesarean… I go on there, a couple of, like, 5- or 10-minute bouts a day, just to do some sort of running exercise now.” (Woman 6) *

With psychological capability, enablers mentioned by women included having knowledge about healthy eating during pregnancy (2 women), receiving information about managing borderline GDM (2 women), and gaining awareness about GDM (1 woman). Sources for women to obtain relevant information included television, radio, magazines, family members, and printed materials received through the IDEAL study.

*“I suppose having information to start with, having these booklets (from the IDEAL study) easy to read, and filling out your own plan, made you think about those things.” (Woman 15)*

*“I think just, awareness, sort of knowing what you have to do, like, you just don't want to do the wrong thing.” (Woman 20)*

*Opportunity. *Social and physical opportunities were identified under this category. The only social enabler mentioned was support and/or encouragement from family members and friends (8 women). Physical opportunities identified included the baby being active which offered more opportunity to move around (1 woman), baby being easy to look after which allowed for more time (1 woman), affordable childcare at gyms (1 woman), exercise sessions available on weekends (1 woman), and being off work (1 woman).

*“I mean my parents are very much…into… encouraging,…we were brought up in an environment of… I would say healthy eating,…, like balanced eating, and being aware of low GI [glycaemic index] and other things…” (Woman 15)*

*Motivation. *For automatic motivation, enablers highlighted included always maintaining a healthy diet and/or active lifestyle (8 women), craving for healthier food (1 woman), and liking exercise (1 woman).

*“Well, actually during the pregnancy itself I was just craving healthier food.” (Woman 10)*

For reflective motivation, the willingness to improve women's health and/or that of their unborn baby or health of the baby had after s/he arrived was the most frequently mentioned enabler (15 women), specifically, care about their own health (11 women), care about the health of unborn baby (6 women), and care about the health of baby after s/he had been born (4 women). Other enablers mentioned included wanting to lose weight or not gaining too much weight (3 women), trying to avoid certain food (e.g., sugar, soft drink) which was thought to be the cause of hyperglycemia (1 woman), trying to set good examples for children at home (1 woman), thinking about and planning diet and lifestyle goals in advance by using booklets from the IDEAL study (1 woman), attending education sessions to discuss goals for diet and exercise (1 woman), and wanting to make good value of the money paid for exercise sessions (1 woman).

*“Probably just prioritised, I don't want to put baby at risk of gestational diabetes, so you know, make sure I do what I need to do to keep her healthy.” (Woman 17) *

*“I wanna try (to) lose heaps more weight, cos after I had the other baby I put heaps of weight on, this time trying to lose like, heaps more and then try to, just, be fit.” (Woman 12)*

#### 3.6.2. Barriers Identified by Women

*Capability.* With physical capability, seven women mentioned being “tired,” “exhausted,” or “no energy” as their barriers to achieving their intended diet and exercise goals. Tiredness was raised as a barrier by both antenatal women and postnatal women. For antenatal women, the tiredness was more frequently related to pregnancy itself, while postnatal women's tiredness was more likely to be a result of breastfeeding on demand and not having enough sleep. Other barriers reported by women included “experiencing a painful pregnancy,” “feeling sick and nauseous,” “low sitting placenta,” “having caesarean section,” “feeling unwell,” and “knee problem.”

*“…getting up with her during the night, I was very tired, and I'd kind of just ate a lot of sugar to give me energy.” (Woman 9)*

*“Because I can't, like cook every day, it's very tiring; so basically, I normally will have (to) go outside about 2-3 times a week. This is only main problem.” (Woman 22)*

In terms of psychological capability, the barriers reported included being unsure about diet and exercise recommendations for women with borderline GDM (1 woman) and the belief that pregnant women should not start exercising if they were not active before pregnancy (1 woman).

*Opportunity. *For physical opportunity, being too busy and/or lack of family support were the most frequently mentioned barriers (7 women). Other mentioned barriers were sugary food or chocolate being easily accessible (4 women), bad weather or getting dark early during winter (2 women), changing in climate and environment while moving to another country (1 woman), shopping with kids (1 woman), and having meals away from home (1 woman).

For social opportunity, the perceptions that new mums could have chocolate, cakes, or something sweet (1 woman) and lack of support from family members were raised.

*Motivation.* Automatic motivation was the only theme identified under this category. The barriers mentioned included not being highly motivated (3 women), personal preference (2 women), habits (2 women), and pregnancy craving (1 woman).

*“Maybe just like, I'm already fat or heavier after I give birth… Just leave it.” (Woman 11)*

*“…you know just crave for something like that (chocolate).” (Woman 15)*

### 3.7. Women's Needs to Overcome Barriers

The needs expressed by women during their interviews varied considerably, depending on the barriers they experienced (Table 4). The most frequently mentioned needs were better family support from partners and/or parents. Two women reported that nothing could help, as the barriers expressed by these two women were food craving and tiredness relating to pregnancy itself. Three of the four women who did not plan any changes to their diet or exercise also expressed their needs as receiving family support (2 women), having information about nutrition and/or management for borderline GDM (2 women), overcoming depression (1 woman), educating people around about nutrition for pregnant women (1 woman), and being able to organize their time better (1 woman).

## 4. Discussion

From our face-to-face, semistructured interview study, we find that a diagnosis of pregnancy hyperglycemia without meeting GDM diagnostic criteria causes concerns for some women. Managing this mild form of pregnancy hyperglycemia is perceived by women as important, although most women do not seek out information by themselves. Women are willing to improve their lifestyle but achieving a successful lifestyle modification is influenced by a wide range of factors. Thinking about baby's health and their own health was highlighted as one of the most important facilitators to achieve a healthier lifestyle. On the other hand, being physically unwell, having a busy life, and not having adequate family support were the most frequently mentioned inhibitors. Women with pregnancy hyperglycemia express many different needs, the most common being need for better family support and receiving appropriate diet and exercise information.

Previous studies using a semistructured or in-depth interview method have been undertaken to examine women's experiences and attitudes after being diagnosed with GDM and the facilitators and inhibitors to the intended lifestyle management [23–30]. However, there are limited data on women's experiences after being diagnosed with pregnancy hyperglycemia without meeting GDM diagnostic criteria and little is known about the enablers and barriers for them to achieve healthier lifestyles.

Findings from qualitative studies targeting women with GDM (meeting GDM diagnostic criteria) [23–30] provide a context for our results although their study populations are different from those of the current study, as the women have greater degrees of pregnancy hyperglycemia.

In contrast with our results, negative feelings such as being upset, fear, shock, or worries after the diagnosis of GDM were more frequently mentioned in previous studies investigating the experience of women with GDM [24–28, 30].

Consistent with our results, concerns about baby's health were found as a main motivational factor for women seeking GDM management [23, 25, 29, 30] and more information about lifestyle management after diagnosis was wanted [28].

Time pressures, physical constraints, and lack of clear guidelines were the main barriers to achieving lifestyle self-management. Facilitators in other studies were thinking about the baby and having support from family members in women with GDM from low socioeconomic and migrant backgrounds living in Australia [23]. These findings are similar to our study results. Amongst 17 immigrant women from South Asia with GDM living in Australia, the need for culturally appropriate dietary advice was found [27]. This was not apparent in our study, perhaps because we only included English-speaking women in our study.

We believe our study to be the first face-to-face, semistructured interview study targeting women with borderline GDM. It helps provide an in-depth understanding of women's views and perceptions towards the diagnosis and management of borderline GDM, as well as providing information about important factors that affect women's ability to achieve their intended lifestyle modifications. Therefore, the findings of our study may help with designing and providing tailored care for women with mild pregnancy hyperglycemia in the future. A limitation to our study is that only women who could speak English were eligible, so women from different cultural backgrounds may have been excluded. Inclusion of non-English speaking women from different ethnic groups may be worth considering in future studies, given our culturally diverse community in Australia. Our findings are based on information provided by a relatively small number of women from one geographical area, which may have limited generalizability to pregnant populations. Although some participants were interviewed antenatally and some postnatally, this spread over different gestational stages helps generalizability of the study findings.

## 5. Conclusions

This study shows the diagnosis of borderline GDM can cause worries for some women although lifestyle management was identified as important by most women affected. Factors impacting women's ability to achieve intended lifestyle changes vary greatly, with the most important enabler being thinking about baby's health and their own health and the most significant barrier being a lack of family support.

## Figures and Tables

**Table 1:** Characteristics of women approached for the study.

Characteristics	Attended interview		Declined interview		Total	
	*N* = 22^1^		*N* = 3		*N* = 25	
Maternal age (years)^†^	30.3	6.0	31.3	12.5	30.4	6.7
Primiparity	15	68.2	3	100.0	18	72.0
Ethnicity						
(i) Caucasian	13	59.1	3	100.0	16	64.0
(ii) Asian	3	13.6	0		3	12.0
(iii) Others	6	27.3	0		6	24.0
BMI at first visit (kg/m^2^)^‡^	23.3	21.9, 29.3	20.9	20.2, 25.6	23.9	20.6, 28.8
BMI group^§^						
(i) Underweight	1	4.8	0		1	4.2
(ii) Normal	11	52.4	2	66.7	13	54.2
(iii) Overweight	4	19.0	1	33.3	5	20.8
(iv) Obesity	5	22.8	0		5	20.8
Weight at 1st antenatal visit (kg)^†^	67.7	17.0	58.8	2.5	66.6	16.1
Smoker	1	4.5	2	66.7	3	12.0
Maternal history of hypertension	1	4.5	0		1	4.0
Family history of hypertension^*^	6	27.3	0		6	24.0
Family history of diabetes^*^	10	45.5	0		10	40.0
Socioeconomic status^**^						
(i) Most disadvantaged	5	22.7	0		5	20.0
(ii) Disadvantaged	1	4.6	0		1	4.0
(iii) Average	7	31.8	0		7	28.0
(iv) Advantaged	6	27.3	2	66.7	8	32.0
(v) Most advantaged	3	13.6	1	33.3	4	16.0

Figures are number and percentage.

^1^11 women attended interview antenatally and 11 women attended interview postnatally.

^†^Mean and standard deviation; ^‡^median and interquartile range.

^§^Weight and BMI at first antenatal visit were unknown for one woman who attended interview. Underweight: BMI < 18.5 kg/m^2^; normal: BMI 18.5–24.9 kg/m^2^; overweight: BMI 25.0–29.9 kg/m^2^; obesity: BMI ≥ 30 kg/m^2^.

^*^Family history of hypertension and diabetes was unknown for one woman who did not attend interview.

^**^As measured by the Australian Bureau of Statistics Socioeconomic Indexes for Areas [16].

BMI: body mass index.

**Table 2:** Women's experience after being told they had borderline GDM.

Experience	Women	
	Number	%
Not surprised/not worried/felt ok	14	64
Mildly concerned/mildly worried	5	23
Scared/worried/concerned	3	13

GDM: gestational diabetes mellitus.

**Table 3:** Enablers and barriers for women to achieve their lifestyle goals.

Enablers	Capability	Physical	(i) Physical fitness improved over time
		Psychological	(i) Knowing about healthy eating during pregnancy
			(ii) Aware/informed about bGDM/GDM
	Opportunity	Physical	(i) Active baby increases mother's activity
			(ii) Baby, easy to look after, allows more time for healthier lifestyle
			(iii) Affordable childcare at gyms
			(iv) Exercise sessions available on weekends
			(v) Allowed more time while on leave from work
		Social	(i) Support and/or encouragement from family members and friends
	Motivation	Automatic	(i) Used to healthy dietary pattern and/or active lifestyle
			(ii) Craved healthier food
			(iii) Likes exercise
		Reflective	(i) Cared about baby's health and/or own health
			(ii) Wanted to lose weight or not gaining too much weight
			(iii) Tried to avoid food (e.g. sugar, soft drink) “causing” hyperglycemia
			(iv) Tried to set good examples for children at home
			(v) Thought about and planned diet and lifestyle goals in advance by using booklets from research study
			(vi) Attended education sessions to discuss goals for diet and exercise
			(vii) Wanted to make good value of the money paid for exercise sessions

Barriers	Capability	Physical	(i) Being tired and exhausted or having no energy
			(ii) Experienced a painful pregnancy
			(iii) Felt sick and nauseous or unwell
			(iv) Low lying placenta
			(v) Had caesarean section
			(vi) Had knee problem
		Psychological	(i) Unsure about proper diet and lifestyle for women with bGDM
			(ii) Believed that pregnant women should not start exercising if not active before pregnancy
	Opportunity	Physical	(i) Being too busy
			(ii) Lack of family support
			(iii) Bad weather or getting dark early during winter
			(iv) Having easy access to sugary food or chocolate
			(v) Change in weather and environment while moving to another country
			(vi) Shopping with young children is difficult
			(vii) Having meals away from home
		Social	(i) The belief that “new mums could have chocolate, cakes or something sweet”
			(ii) Lack of support from family members
	Motivation	Automatic	(i) Personal preference
			(ii) Habits
			(iii) Craved unhealthy food
			(iv) Not motivated to exercise

GDM: gestational diabetes mellitus; bGDM: borderline gestational diabetes mellitus.

**Table 4:** Summary of needs raised by women to help with overcoming barriers.

Needs	Number of women
Family support from partner and/or parents	5
Diet and exercise information for pregnant women/bGDM	4
Being off work	3
Having diet and/or exercise sessions with health professional	3
Better weather for exercise	2
Educate people around about nutrition for pregnant women	2
Baby sleeps through night/becomes easier to be looked after	2
Access to preprepared healthy food	2
Making own decision on what to eat	1
Access to flexible time childcare	1
Return to normal health after childbirth	1
Help from health professionals to be more motivated	1
Nothing could help	2

bGDM: borderline gestational diabetes mellitus.
